# Efficacy and safety of PD-1 and PD-L1 inhibitors combined with chemotherapy in randomized clinical trials among triple-negative breast cancer

**DOI:** 10.3389/fphar.2022.960323

**Published:** 2022-09-16

**Authors:** Yihang Qi, Wenxiang Zhang, Ray Jiang, Olivia Xu, Xiangyi Kong, Lin Zhang, Yi Fang, Jingping Wang, Jing Wang

**Affiliations:** ^1^ Department of Breast Surgical Oncology, National Cancer Center/National Clinical Research Center for Cancer/Cancer Hospital, Chinese Academy of Medical Sciences and Peking Union Medical College, Beijing, China; ^2^ Department of Anesthesia, Critical Care and Pain Medicine, Massachusetts General Hospital, Harvard Medical School, Boston, MA, United States; ^3^ Department of Neurology, New York University Grossman School of Medicine, New York City, NY, United States; ^4^ School of Population Medicine and Public Health, Chinese Academy of Medical Sciences and Peking Union Medical College, Beijing, China; ^5^ Melbourne School of Population and Global Health, The University of Melbourne, Parkville, VIC, Australia; ^6^ Centre of Cancer Research, Victorian Comprehensive Cancer Centre, Melbourne, VIC, Australia

**Keywords:** breast cancer, immunotherapy, PD-1/PD-L1 immune checkpoint inhibitors, chemotherapy, adjuvant therapy, neoadjuvant therapy

## Abstract

**Background:** The combination of immune checkpoint inhibitors (ICIs) and chemotherapy (CT) is a new strategy to explore cancer treatment in recent years, and it is also practiced in triple-negative breast cancer (TNBC). However, several published randomized controlled trials (RCTs) reported heterogeneous results. We conducted this meta-analysis to yield insights into the efficacy and safety of the combination of ICIs and CT for TNBC patients in both the adjuvant and neoadjuvant settings.

**Method:** EMBASE, PUBMED, Cochrane, and www.clinicaltrials.gov databases were searched to determine potential eligible studies from the inception to 20 May 2022. Published RCTs on PD-1/PD-L1 ICIs combined with CT for TNBC patients were included.

**Result:** This meta-analysis included six double-blind RCTs comprising 4,081 TNBC patients treated with PD-1 or PD-L1 ICIs plus CT or placebo plus CT. The combination strategy benefited a better pathologic complete response (pCR) by 29% (RR = 1.29; 95% CI: 1.17–1.41; I^2^ = 0%) and a better progression-free survival (PFS) (HR = 0.82; 95% CI: 0.74–0.90; I^2^ = 0%) in the neoadjuvant and the adjuvant settings, respectively, especially in PD-L1-positive population (HR = 0.71; 95% CI: 0.62–0.81; I^2^ = 13%). The safety profiles were generally tolerable in both settings but the combination treatment will increase the risk of severe adverse events in the adjuvant setting (RR = 1.33; 95% CI 1.08–1.62, I^2^ = 0%). Additionally, the combination will increase the risk of any-grade hypothyroidism, hyperthyroidism, pneumonia, and rash in the adjuvant setting, and the risk of any-grade hypothyroidism, hyperthyroidism, infusion-related reactions, and severe cutaneous reactions in the neoadjuvant setting.

**Conclusion:** This meta-analysis demonstrated a significant pCR benefit and confirms the PFS benefit with PD-1/PD-L1 ICIs plus CT in TNBC patients with tolerable safety events in both neoadjuvant and adjuvant settings.

## Introduction

Triple-negative breast cancer (TNBC) stands as a particularly aggressive type of cancer with a relatively poor prognosis. TNBC indicates the presence of cancerous cells that screen negative for three molecular constituents of breast cancer: estrogen receptors (ERs), progesterone receptors (PRs), and sufficient levels of human epidermal growth factor (HER2) protein (Anders et al., 2016). Due to the molecular characteristics of TNBC, limited target therapies exist, and chemotherapy (CT) consequently remains one of the only options for treatment (Adel, 2021). Around 10%–20% of all breast cancer cases are composed of TNBC cases (Adel, 2021), and individuals with a breast cancer gene 1 (BRCA1) mutation have an increased genetic susceptibility to developing the cancer (Anders et al., 2016). BRCA1 is a tumor suppressor gene that plays an essential role in regulating DNA damage through homologous recombination-mediated repair of double-strand breaks (Anders et al., 2016). Thus, when mutated, pathogenic variants of this gene can lead to the formation of metastatic tumors that are defective in this method of DNA repair, resulting in cases of TNBC.

One molecular attribute that is often indicative of cancer, is the ability of cells to evade immune response through various pathways such as the PD-1/PDL-1 pathway. Programmed death protein 1 (PD-1) is an inhibitory cell surface receptor that binds to programmed death-ligand 1 (PD-L1), and activates a downstream signaling cascade that inhibits T-cell activation, and represses the immune system’s ability to initiate any inflammatory response (Ali et al., 2019). When PD-1 is overexpressed in cancerous cells, cells can evade the immune response and proliferate uncontrollably, consequently resulting in various types of cancer including TNBC (Anders et al., 2016). To control this rapid growth and spread, anti-PD-1/PD-L1 immune checkpoint inhibitors (ICIs) can be introduced to prevent the binding interaction between PD-1/PD-L1 (Ali et al., 2019), thereby generating a positive immune response that can eliminate cancerous cells.

The efficacy and use of immuno-monotherapy for TNBC patients have not yet been defined (Anders et al., 2016). However, ICIs can be used in conjunction with CT to target and treat aggressive tumors, and further exploration in regards to this combinatorial treatment has led to more promising results. Various RCTs have recently surfaced, analyzing different ICI/CT treatments in patients diagnosed with metastatic TNBC (Villacampa et al., 2022). These studies demonstrate that treatment of ICIs in conjunction with CT can be applied to adjuvant (administered after primary treatment) and neoadjuvant (administered before primary treatment) therapies, both of which may yield optimal results. Six specific RCTs analyzed in this review include three studies involving adjuvant treatment [IMpassion131 (Miles et al., 2021), IMpassion130 (Adams et al., 2020), and KEYNOTE-355 (Cortes et al., 2020)], and three studies involving neoadjuvant treatment [IMpassion031 (Mittendorf et al., 2020), KEYNOTE-522 (American Association for Cancer Research, 2019), and GeparNuevo (Loibl et al., 2019)]. Overall, this review aimed to compare combinatorial treatment options (ICIs + CT), along with traditional CT treatments, to examine if the former yields more tolerable adverse events and better efficacies in treating TNBC patients.

## Methods

A meta-analysis together with a systematic review was performed to enroll RCT studies concerning the combination strategy of PD1/PD-L1 ICIs plus CT compared with CT monotherapy in TNBC patients applied whether as adjuvant therapy or neoadjuvant therapy. The meta-analysis was conducted according to the Preferred Reporting Items for Systematic Reviews and Meta-Analyses (PRISMA) guidelines [20].

### Literature searches

We searched EMBASE, PUBMED, Cochrane, and www.clinicaltrials.gov databases to enroll potential eligible studies from the inception to 20 May 2022. We performed the searching using the following strategy: [“nivolumab” OR “pembrolizumab” OR “atezolizumab” OR “avelumab” OR “BMS-936559″ OR “durvalumab” OR (“pd l1 inhibitors” OR “pd l1 inhibitors” OR “pd l1 inhibitor” OR “pd l1 inhibitor” OR “programmed death ligand 1 inhibitors” OR “programmed death ligand 1 inhibitors” OR “pd 1 inhibitors” OR “pd 1 inhibitors” OR “pd 1 inhibitor” OR “inhibitor pd 1″ OR “pd 1 inhibitor” OR “programmed cell death protein 1 inhibitor” OR “programmed cell death protein 1 inhibitors” OR “pd 1 pd l1 blockade” OR “blockade pd 1 pd l1″ OR “pd 1 pd l1 blockade” OR “Immune Checkpoint Inhibitors")] AND {[(“ER-Negative” AND “PR-Negative” AND “HER2-Negative”) AND “breast neoplasms”] OR {[(“eye rep” OR “expert rev mol med” OR “educ res” OR “econ rec” OR “ER”) AND (“Negative” OR “negatively” OR “negatives” OR “negativities” OR “negativity”) AND (“psychopathol rev” OR “pharmacol rep” OR “pharmacognosy res” OR “partis rev” OR “PR”) AND (“Negative” OR “negatively” OR “negatives” OR “negativities” OR “negativity”) AND “HER2″] AND “negative breast neoplasms”} OR “triple negative breast cancer” OR “breast cancer triple negative” OR “breast cancers triple negative” OR “triple negative breast cancers” OR “triple negative breast neoplasm” OR [(“breast neoplasms” OR (“Breast” AND “Neoplasms”) OR “breast neoplasms” OR (“Breast” AND “Neoplasm”) OR “breast neoplasm”] AND “Triple-Negative”) OR “breast neoplasms triple negative” OR “triple negative breast neoplasm” OR “Triple Negative Breast Neoplasms” OR “er negative pr negative her2 negative breast cancer” OR “er negative pr negative her2 negative breast cancer” OR “triple negative breast cancer” OR “Triple Negative Breast Neoplasms”}. Available Medical Subject Headings (MeSH) in our strategy were searched by MeSH. The relevant references and reviews were either retrieved to acquire potential studies. Abstracts of conferences posted before 20 May 2022, were also reviewed.

### Selection criteria

The following criteria should be considered and met for the enrolled eligible studies: 1) RCTs concerning the combination therapy of ICIs and CT compared with CT monotherapy; 2) should be applied whether as adjuvant therapy or neoadjuvant therapy; 3) in TNBC patients; 4) RCTs with efficacy outcomes assessed by a hazard ratio (HR) and confidence intervals (CI); 5) adverse events (AEs) and response rate (assessed by the WHO criteria) were analyzed and released.

Exclusion criteria are as follows: 1) non-RCT studies such as single-arm trials and retrospective studies; 2) studies that did not apply placebo plus CT as the strategy of the control group; 3) studies that did not use a PD-1/PD-L1 ICIs plus CT treatment in the experiment group; 4) ongoing clinical trials without released results at the time of the literature search; 5) reviews, systematic reviews, basic research, case reports, meta-analysis, letters, editorials, and expert opinions; 6) unpublished or duplicated studies.

### Data extraction

The titles and abstracts of all screened studies were reviewed by two authors, namely, YQ and RJ independently. The full texts were subsequently assessed for potentially eligible studies. A standardized piloted form was applied to retrieve information from the enrolled studies.

The following variables were retrieved: name of the studies, first author, publication year, study design, endpoint, blinding status, study phase, lines of treatment, median follow-up time, population characteristics, study sample size, experimental group sample size, control group sample size, ICIs used in combination with CT as an experimental arm, CT regimen used as a control arm, biomarker and PD-L1-positive definition assays, intention-to-treat population, PD-L1 status subgroups, safety population sample size; ECOG performance status, median age, female percentage, race percentage, number of metastatic sites, previous neoadjuvant/adjuvant CT, stage IV at initial diagnosis, frequency of pCR events in neoadjuvant studies, HR with associated 95% CI for PFS and OS, frequency of AEs of any grade reported, grade ≥3 AEs, and severe adverse events (SAEs) for the experimental group. Adverse events are graded by 1–5, and recorded according to Version 4 of the Common Terminology Criteria for Adverse Events of the National Cancer Institute, CTC for AE version. The discrepancies were discussed thoroughly and well-solved.

### Quality assessment

The risk of bias was discussed and assessed according to the Cochrane Collaboration’s Risk of Bias tool by two independent investigators. We assessed each study’s risk of bias according to exclusion criteria, study design, and observation period considerations.

### Outcomes

The primary endpoints are as follows: 1) pCR rate in neoadjuvant therapy studies, defined as pathological complete response with no cancer cells existing in the pathological examination of cancer patients after treatment; 2) PFS in adjuvant therapy studies, which was defined as the time from the date of randomization to the date of the first record of disease progression (according to RECIST 1.1) or any-cause death. The secondary efficacy endpoint was OS, which was defined as the time from randomization to any-cause death. Safety endpoints are 1) percentage of AEs of any grade, 2) grade ≥3 AEs, and 3) severe AEs.

### Statistical analysis

PFS and OS were analyzed by calculating HR with 95% CI to summarize the efficacy. For the pCR rates and safety assessment, RR with 95% CI was determined to obtain an overall estimation. HR < 1 indicates a protective effect with ICIs plus CT, while an RR > 1 indicates a higher possibility of adverse events for patients treated with the combination therapy.

The Q test and I^2^ statistics were performed to assess heterogeneity between included studies. The meta-analysis was performed using the fixed-effects model only if the I^2^ value was less than 50%, otherwise, the random-effects model will be selected. Otherwise, the random-effects model was selected. The Egger’s test and funnel plot were conducted to examine the potential publication. All analyses were performed using R statistical software version 3.6.2 (R packages metafor and meta).

## Results

### Literature search

**FIGURE 1:**
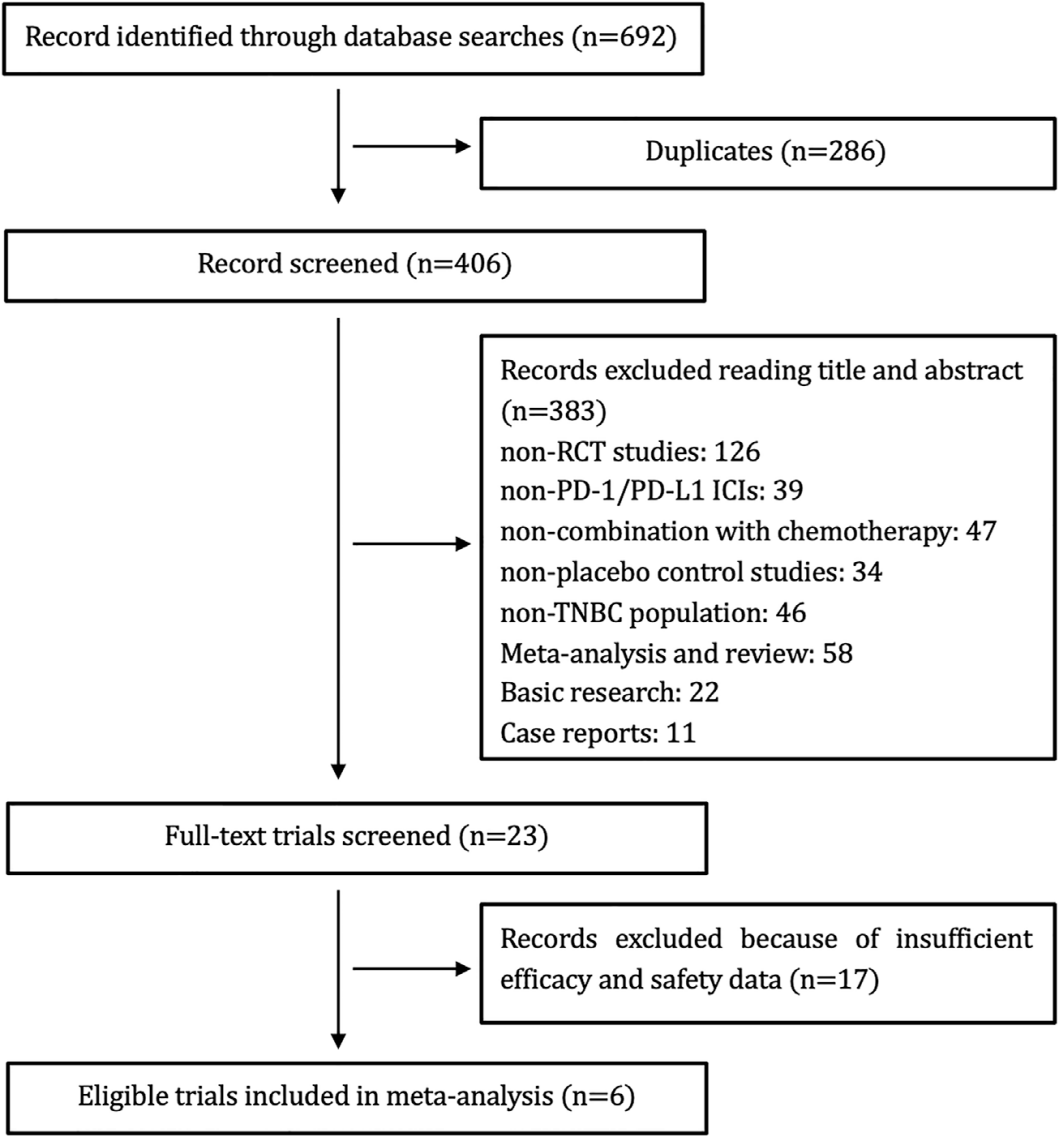
Flow diagram for identification and selection of studies included in the meta-analysis.

We identified 692 potential articles, 286 studies of which were excluded due to duplications. We screened the titles and abstracts of the remaining 406 articles, 383 of which were again removed according to our inclusion or exclusion criteria. An additional of 17 studies were excluded because they did not contain our data of interest. Ultimately, six studies were pooled for our meta-analysis. The study selection diagram is shown in Figure 1.

### Study characteristics

The study design and baseline information on the involved clinical trials are demonstrated in Table 1. We included six double-blind RCTs comprising 4,081 TNBC patients treated with PD-1/PD-L1 ICIs plus chemotherapy or placebo plus chemotherapy in this meta-analysis (Loibl et al., 2019; Cortes et al., 2020; Mittendorf et al., 2020; Schmid et al., 2020; Emens et al., 2021; Miles et al., 2021) (Table 1). Among these six RCTs, three were adjuvant settings in advanced TNBC patients and the other three are neoadjuvant settings in early-stage TNBC patients.

**TABLE 1:** Characteristics of the studies included in the meta-analysis.

Study	Year	Design	Phase	Population characteristics	Control arm	Experimental arm	Sample size	PD-L1-positive subset	Bio-marker cell	PD-L1-positive definition assay
IMpassion131	2021	RCT double-blind	III	Unresectable locally advanced/metastatic triple-negative breast cancer: no prior systemic therapy or ≥12 months since (neo)adjuvant chemotherapy	Placebo (days 1 and 15) + paclitaxel 90 mg/m (2) (days 1, 8, and 15), every 28 days	Atezolizumab 840 mg (days 1 and 15) + paclitaxel 90 mg/m (2) (days 1, 8, and 15), every 28 days	651	292.95 (45%)	PD-L1 IC	Immune cell expression ≥1%, VENTANA PD-L1 (SP142) assay
IMpassion130	2021	RCT double-blind	III	Unresectable, locally advanced, or metastatic TNBC. Patients had to be eligible for taxane monotherapy, have had no previous chemotherapy or targeted therapy for metastatic TNBC	Placebo (days 1 and 15) + nab-paclitaxel 100 mg/m (2) (days 1, 8, and 15), every 28 days	Atezolizumab 840 mg (days 1 and 15) + nab-paclitaxel 100 mg/m (2) (days 1, 8, and 15), every 28 days	902	369.82 (41%)	PD-L1 IC	PD-L1 expression on IC as a percentage of tumor area [<1% (PD-L1 IC negative) versus 1% (PD-L1 IC positive) using the VENTANA SP142 PD-L1 immunohistochemistry assay. (Ventana Medical Systems, Oro Valley, AZ)]
KEYNOTE-355	2020	RCT double-blind	III	Previously untreated locally recurrent inoperable or metastatic triple-negative breast cancer	Placebo–chemotherapy	200 mg of pembrolizumab (Keytruda, Merck Sharp and Dohme) every 3 weeks in combination with one of three chemotherapy options; nab-paclitaxel 100 mg/m^2^ on days 1, 8, and 15, every 28 days; paclitaxel 90 mg/m^2^ on days 1, 8, and 15, every 28 days	847	636.097 (75.1%)	PD-L1 iTILs	By means of the PD-L1 IHC 22C3 pharmDx assay (Agilent Technologies, Carpinteria, CA, United States) and characterized by the CPS, defined as the number of PD-L1-positive cells (tumor cells, lymphocytes, and macrophages) divided by total number of tumor cells × 100.2. PD-L1-positive tumors are classified as CPS of 1 or more and CPS of 10 or more, and PD-L1-negative tumors are classified as CPS less than 1
IMpassion031	2020	RCT double-blind	III	Early-stage triple-negative breast cancer	Placebo every 2 weeks. Chemotherapy comprised nab-paclitaxel at 125 mg/m^2^ every week for 12 weeks followed by doxorubicin at 60 mg/m^2^ and cyclophosphamide at 600 mg/m^2^ every 2 weeks for 8 weeks, which was then followed by surgery	Chemotherapy plus intravenous atezolizumab at 840 mg or placebo every 2 weeks. Chemotherapy comprised nab-paclitaxel at 125 mg/m^2^ every week for 12 weeks followed by doxorubicin at 60 mg/m^2^ and cyclophosphamide at 600 mg/m^2^ every 2 weeks for 8 weeks, which was then followed by surgery	333	153.846 (46.2%)	PD-L1 IC	PD-L1-positive, that is, patients with PD-L1-expressing tumor infiltrating immune cells covering ≥1% of tumor area
KEYNOTE-522	2020	RCT double-blind	III	Previously untreated stage II or stage III triple-negative breast cancer	Placebo every 3 weeks plus paclitaxel and carboplatin (390 patients). The group then received an additional of four cycles of placebo, and doxorubicin–cyclophosphamide or epirubicin–cyclophosphamide. After definitive surgery, the patients received placebo every 3 weeks for up to 9 cycles	Neoadjuvant therapy with four cycles of pembrolizumab (200 mg) every 3 weeks plus paclitaxel and carboplatin (784 patients). The group then received an additional of four cycles of pembrolizumab, and doxorubicin–cyclophosphamide or epirubicin–cyclophosphamide. After definitive surgery, the patients received adjuvant pembrolizumab every 3 weeks for up to 9 cycles	1,174	972.072 (82.8%)	PD-L1 iTILs	PD-L1 expression in archival or newly obtained formalin-fixed tumor samples was assessed at a central laboratory by means of the PD-L1 IHC 22C3 pharmDx assay (Agilent Technologies). Expression was characterized according to the combined positive score: the number of PD-L1-positive cells (tumor cells, lymphocytes, and macrophages) divided by the total number of tumor cells multiplied by 100; specimens with a combined positive score of 1 or greater were considered PD-L1–positive. Patients were eligible for the trial regardless of PD-L1 status
GeparNuevo	2019	RCT double-blind	II	Early triple-negative breast cancer	Placebo given every 4 weeks in addition to nab-paclitaxel followed by standard EC	Durvalumab given every 4 weeks in addition to nab-paclitaxel followed by standard EC	174	151.902 (87.3%)	PD-L1 iTILs	PD-L1 (n ¼ 158, using Ventana SP263 antibody). We evaluated PD-L1 expression as percentage of tumor cells with membranous staining (PD-L1-TC) and percentage of TILs with membranous or cytoplasmic staining (PD-L1-IC; relative to total TILs). PD-L1 positivity was defined as 1% in one or both percentages

In adjuvant settings (three studies, *n* = 2,400), all three studies are phase III clinical trials, with atezolizumab used in IMpassion 130 and IMpassion 131 (*n* = 1,553; 64.7%) and pembrolizumab used in KEYNOTE-355 (*n* = 847; 35.3%) as immunotherapy agents. Nab-paclitaxel, paclitaxel, and gemcitabine–carboplatin were, respectively, used as CT regimens in different studies. In neoadjuvant settings (three studies, *n* = 1,681), IMpassion 031 using atezolizumab (*n* = 333, 19.8%) and KEYNOTE-522 using pembrolizumab (*n* = 1,174, 69.8%) are phase III clinical trials, and GeparNuevo using durvalumab (*n* = 174, 10.4%) is a phase II study. Among these 4,081 patients, 2,577 patients were PD-L1+ (the positive status was generally defined as PD-L1 expressed in more than 1% tumor cells or immune cells, and the specific PD-L1-positive definition assays are demonstrated in Table 1).

## Efficacy analysis

### Progression-free survival in the adjuvant setting

**FIGURE 2:**
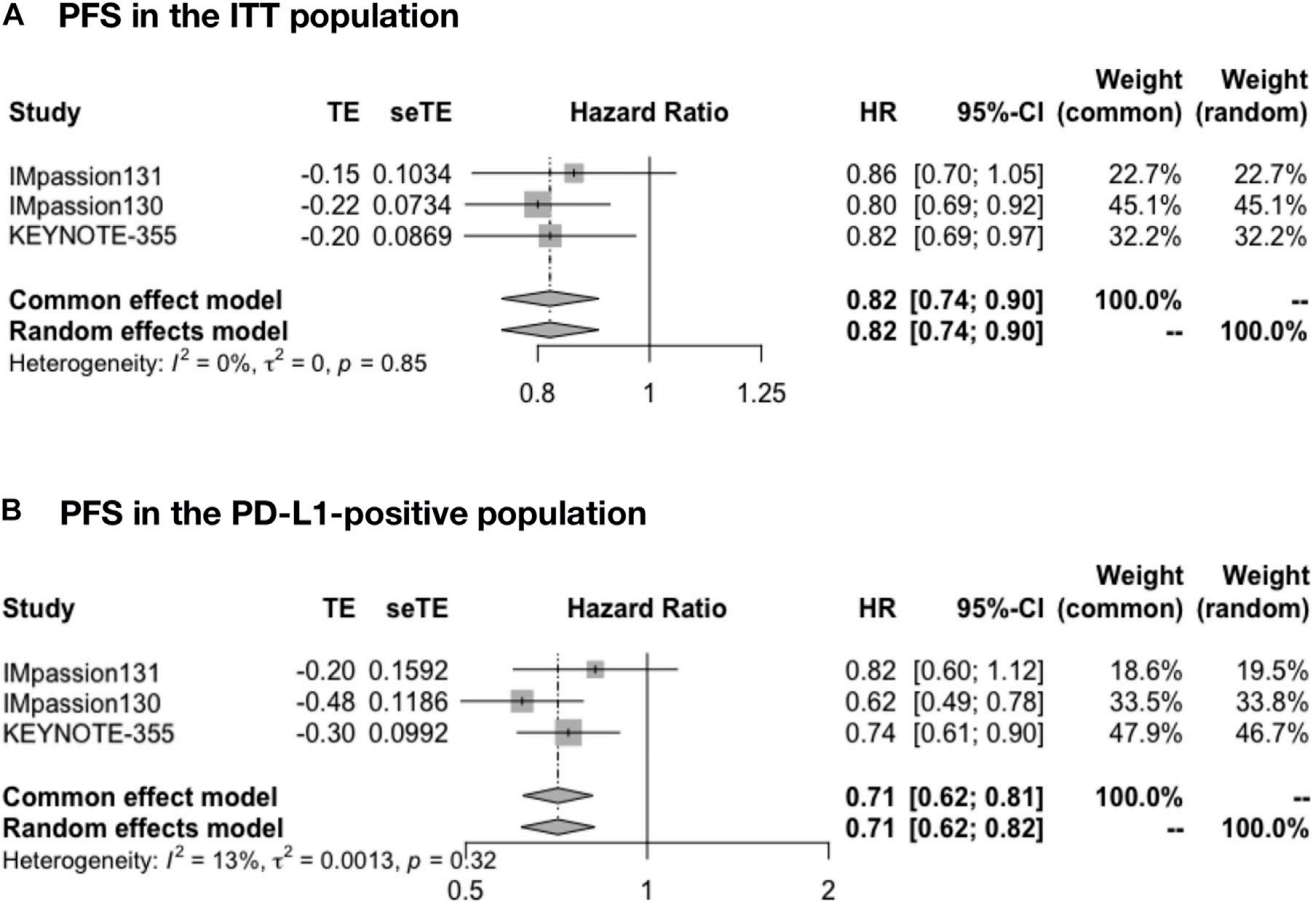
Pooled PFS in adjuvant therapy clinical trials. **(A)** PFS pooled results in the ITT population in the comparison of ICIs plus CT versus placebo plus CT. **(B)** PFS pooled results in the PD-L1-positive population. PFS, progression-free survival; ITT, intent-to-treat; HR, hazard ratio; 95% CI, 95% confidence interval; ICIs, immune checkpoint inhibitors; CT, chemotherapy.

The pooled PFS was analyzed in IMpassion 130, IMpassion 131, and KEYNOTE-355 (*n* = 2,400), the pooled evaluation in the ITT population demonstrated a benefit for the ICIs plus CT group with no heterogeneity was found (HR = 0.82; 95% CI: 0.74–0.90; I^2^ = 0%) (Figure 2A). Furthermore, a significantly better PFS was found in PD-L1-positive population (*n* = 984) in the combination of ICIs and CT regimens (HR = 0.71; 95% CI: 0.62–0.81; I^2^ = 13%) (Figure 2B).

### Overall survival and death risk in the adjuvant setting

**FIGURE 3:**
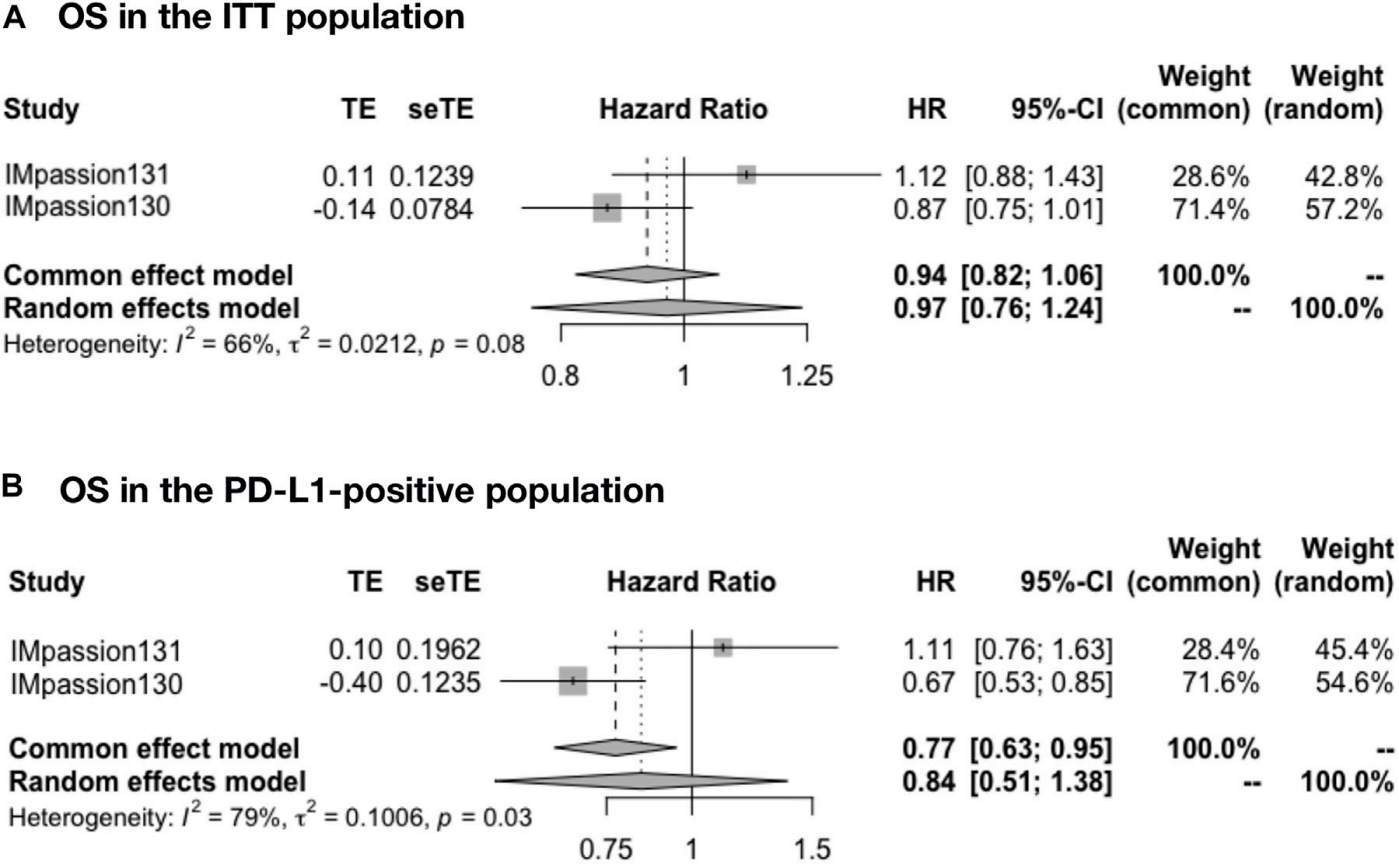
Pooled OS in adjuvant therapy clinical trials. **(A)** OS pooled results in the ITT population in the comparison of ICIs plus CT versus placebo plus CT. **(B)** OS pooled results in the PD-L1-positive population. OS, overall survival; ITT, intent-to-treat; HR, hazard ratio; 95% CI, 95% confidence interval; ICIs, immune checkpoint inhibitors; CT, chemotherapy.

The pooled OS was analyzed in IMpassion 130, IMpassion 131, and KEYNOTE-355 (*n* = 2,400). The pooled OS evaluation demonstrated no benefit of ICIs plus CT with heterogeneity in both the ITT population (HR = 0.97; 95% CI: 0.76–1.24; I^2^ = 66%) (Figure 3A) and the PD-L1-positive population (HR = 0.84; 95% CI: 0.51–1.38; I^2^ = 79%) (Figure 3B).

### Time to deterioration in global health status/health-related quality of life in the adjuvant setting

**FIGURE 4:**
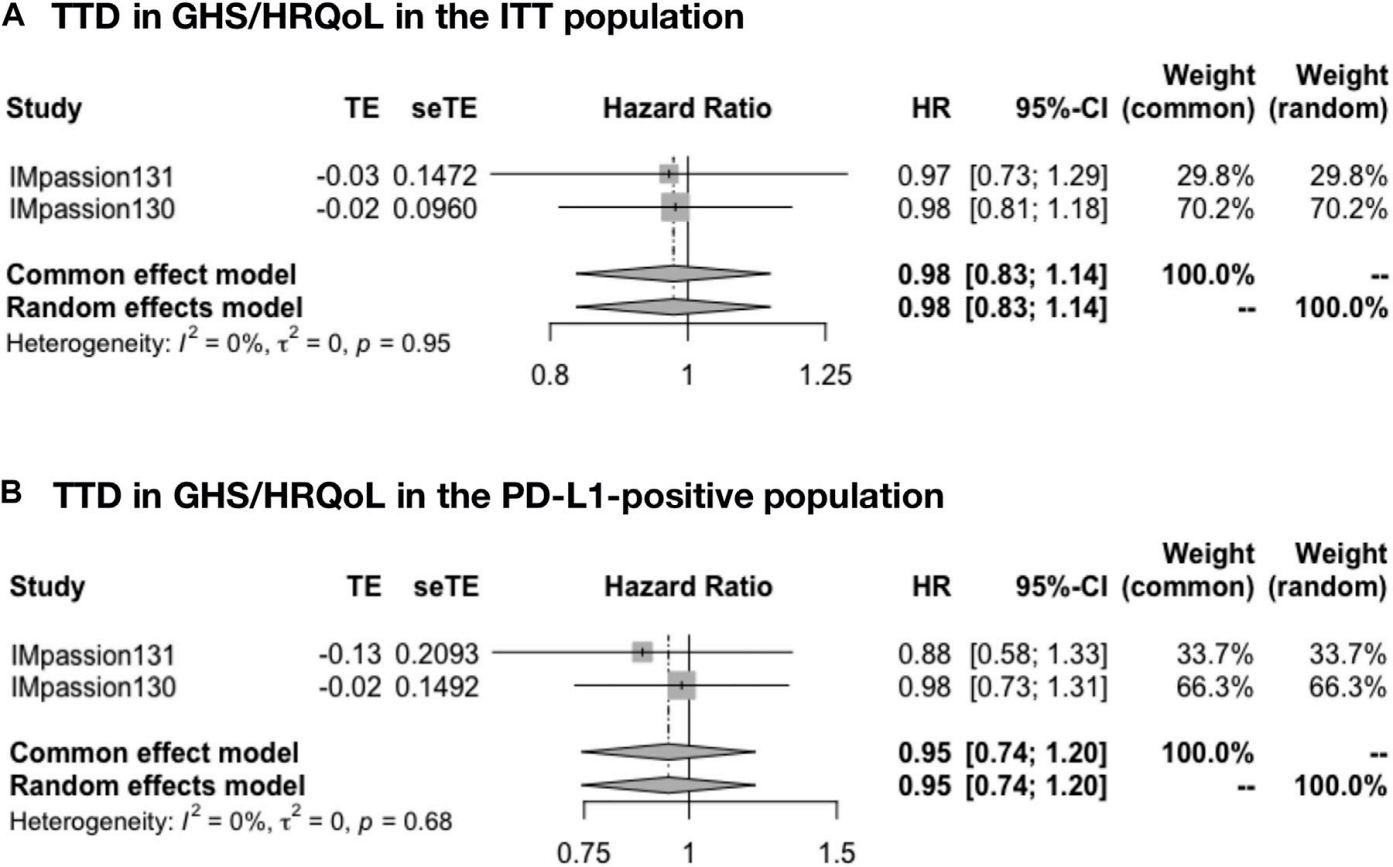
Pooled TTD in adjuvant therapy clinical trials. **(A)** TTD in GHS/HRQoL pooled results in the ITT population in the comparison of ICIs plus CT versus placebo plus CT adjuvant therapy. **(B)** TTD in GHS/HRQoL pooled results in the PD-L1-positive population in the comparison of ICIs plus CT versus placebo plus CT adjuvant therapy. TTD, time to deterioration (defined as a 10-point decrease); GHS/HRQoL, global health status/health-related quality of life; ITT, intent-to-treat; HR, hazard ratio; 95% CI, 95% confidence interval; ICIs, immune checkpoint inhibitors; CT, chemotherapy.

The pooled time to deterioration (TTD) in global health status/health-related quality of life (GHS/HRQoL) was analyzed in IMpassion 131 and IMpassion 130 (*n* = 1,553). The pooled evaluation demonstrated no benefit of ICIs plus CT with no heterogeneity in both the ITT population (HR = 0.98; 95% CI: 0.83–1.14; I^2^ = 0%) (Figure 4A) and the PD-L1-positive population (HR = 0.95; 95% CI: 0.74–1.20; I^2^ = 0%) (Figure 4B).

### Pathologic complete response rate in the neoadjuvant setting

**FIGURE 5:**
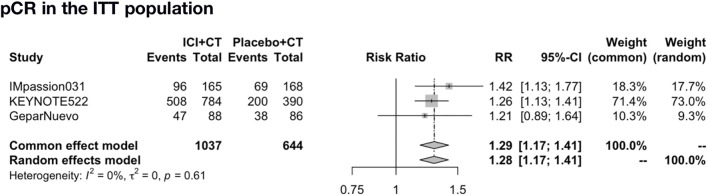
Pooled pCR rate in neoadjuvant therapy clinical trials. pCR pooled results in the ITT population in the comparison of ICIs plus CT versus placebo plus CT neoadjuvant therapy. pCR, pathologic complete response; ITT, intent-to-treat; RR, relative risk; 95% CI, 95% confidence interval; ICIs, immune checkpoint inhibitors; CT, chemotherapy.

The pooled pCR rate was analyzed in IMpassion 031, KEYNOTE-522, and GeparNuevo (*n* = 1,681). The pooled evaluation in the ITT population demonstrated that a significant pCR was in favor of the ICIs plus CT group with no heterogeneity found (RR = 1.29; 95% CI: 1.17–1.41; I^2^ = 0%) (Figure 5).

## Safety analysis

### The overall adverse events in the adjuvant setting

**FIGURE 6:**
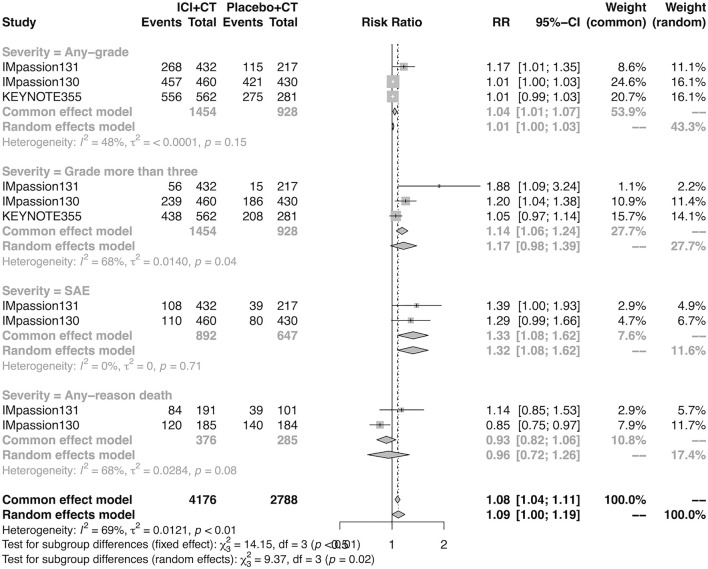
Pooled AEs in adjuvant therapy clinical trials. SAEs, severe adverse events; RR, relative risk; 95% CI, 95% confidence interval; ICIs, immune checkpoint inhibitors; CT, chemotherapy.

Overall, 4,057 of 4,081 (99.4%) patients were included in the safety analysis. The pooled overall AEs in the adjuvant setting was analyzed in IMpassion 130, IMpassion 131, and KEYNOTE-355 (*n* = 2,400). The estimation demonstrated that the combination of ICIs and CT arm was associated with a higher incidence of AEs in any grade with no heterogeneity (RR = 1.04; 95% CI 1.01–1.07, I^2^ = 48%), and a higher incidence of AEs in severe grades with no heterogeneity (RR = 1.33; 95% CI 1.08–1.62, I^2^ = 0%). However, the estimation in the random-effects model showed that no significant variation was found between the experimental arm and control arm in the incidence of AEs more than grade III (RR = 1.17; 95% CI 0.98–1.39, I^2^ = 68%) and any-reason death (RR = 0.96; 95% CI: 0.72–1.26; I^2^ = 68%) (Figure 6).

### The overall adverse events in the neoadjuvant setting

**FIGURE 7:**
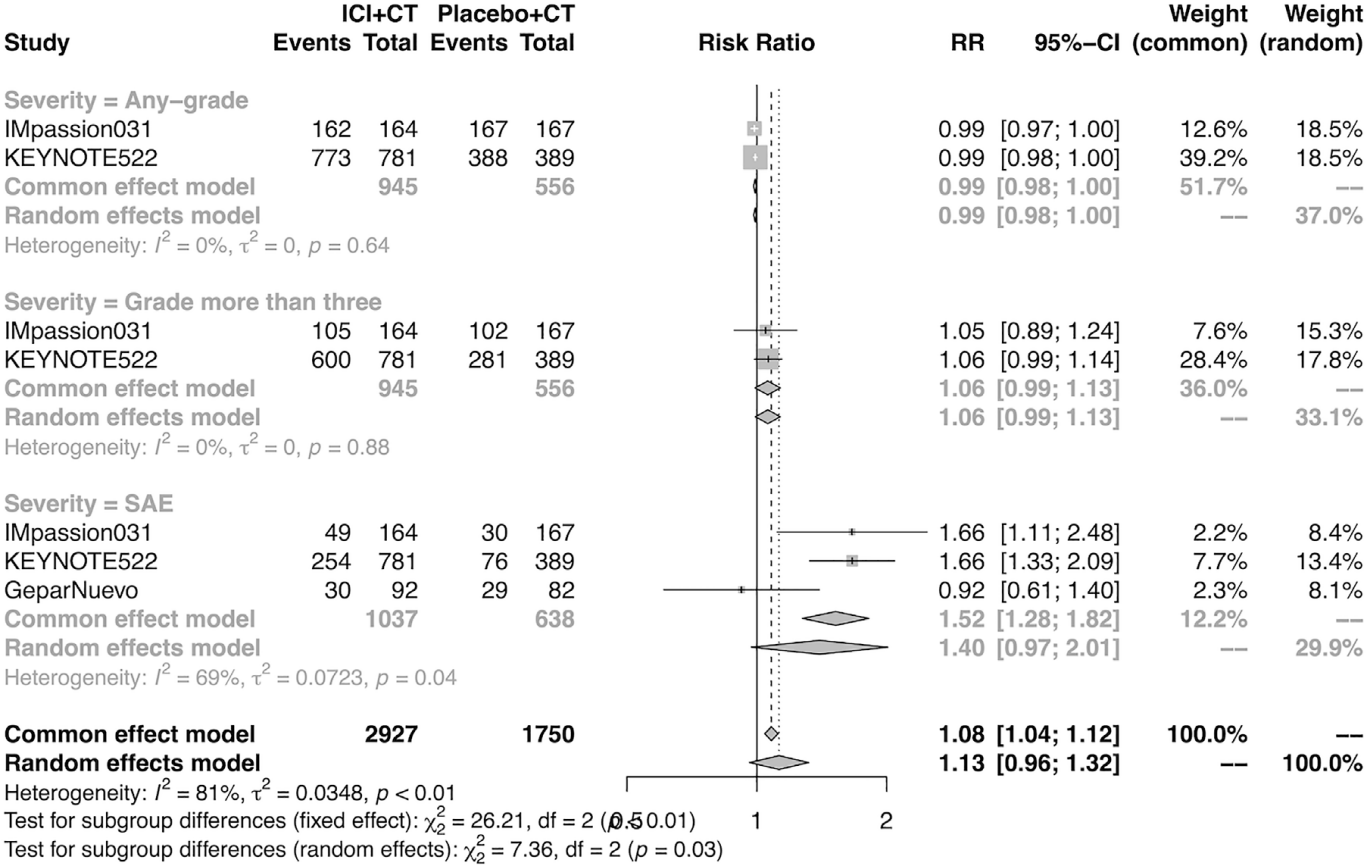
Pooled AEs in neoadjuvant therapy clinical trials. SAEs, severe adverse events; RR, relative risk; 95% CI, 95% confidence interval; ICIs, immune checkpoint inhibitors; CT, chemotherapy.

The pooled overall AEs in the neoadjuvant setting were analyzed in IMpassion 031, KEYNOTE-522, and GeparNuevo. The estimation demonstrated that the combination of ICIs and CT arm was not correspondent with a higher risk of any-grade AEs (RR = 0.99; 95% CI 0.98–1.00, I^2^ = 0%), more than grade III AEs (RR = 1.06; 95% CI 0.99–1.13, I^2^ = 0%) and the SAEs (RR = 1.40; 95% CI 0.97–2.01, I^2^ = 69%) (Figure 7).

### The detail of adverse events in the adjuvant setting

**FIGURE 8:**
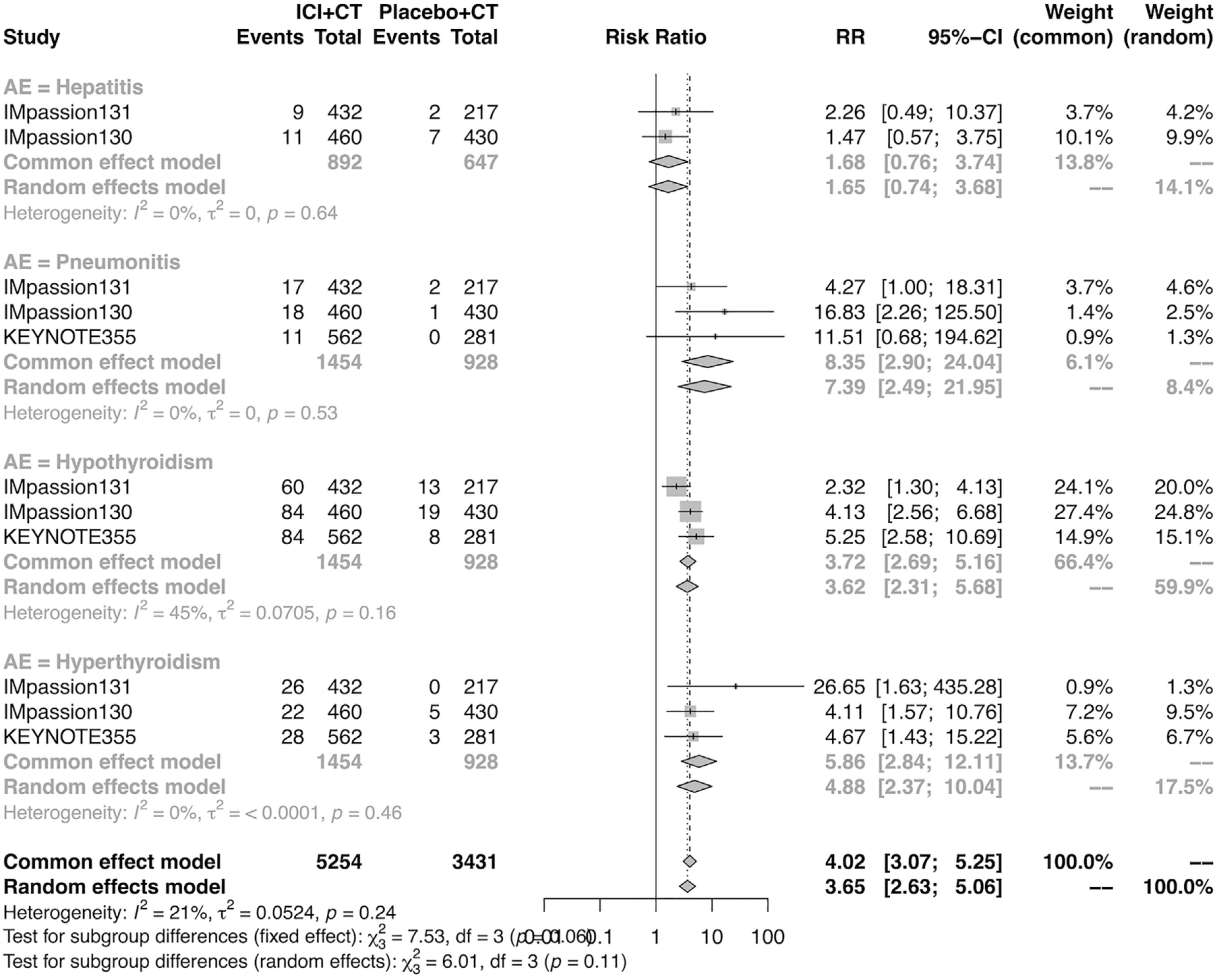
Pooled specific AEs in any grade in adjuvant therapy clinical trials. AEs, adverse events; RR, relative risk; 95% CI, 95% confidence interval; ICIs, immune checkpoint inhibitors; CT, chemotherapy.

**FIGURE 9:**
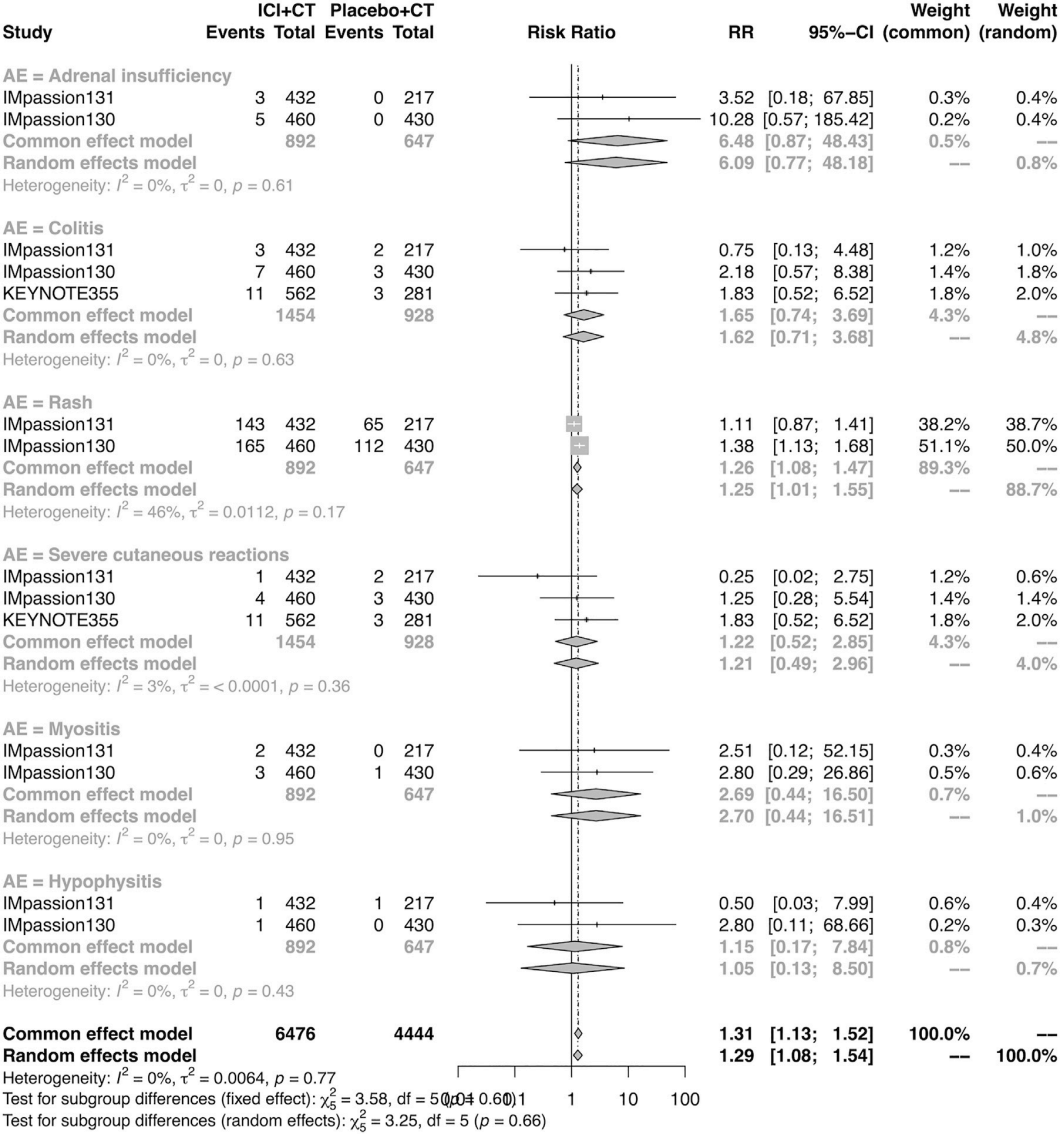
Pooled specific AEs in any grade in adjuvant therapy clinical trials. AEs, adverse events; RR, relative risk; 95% CI, 95% confidence interval; ICIs, immune checkpoint inhibitors; CT, chemotherapy.

**FIGURE 10:**
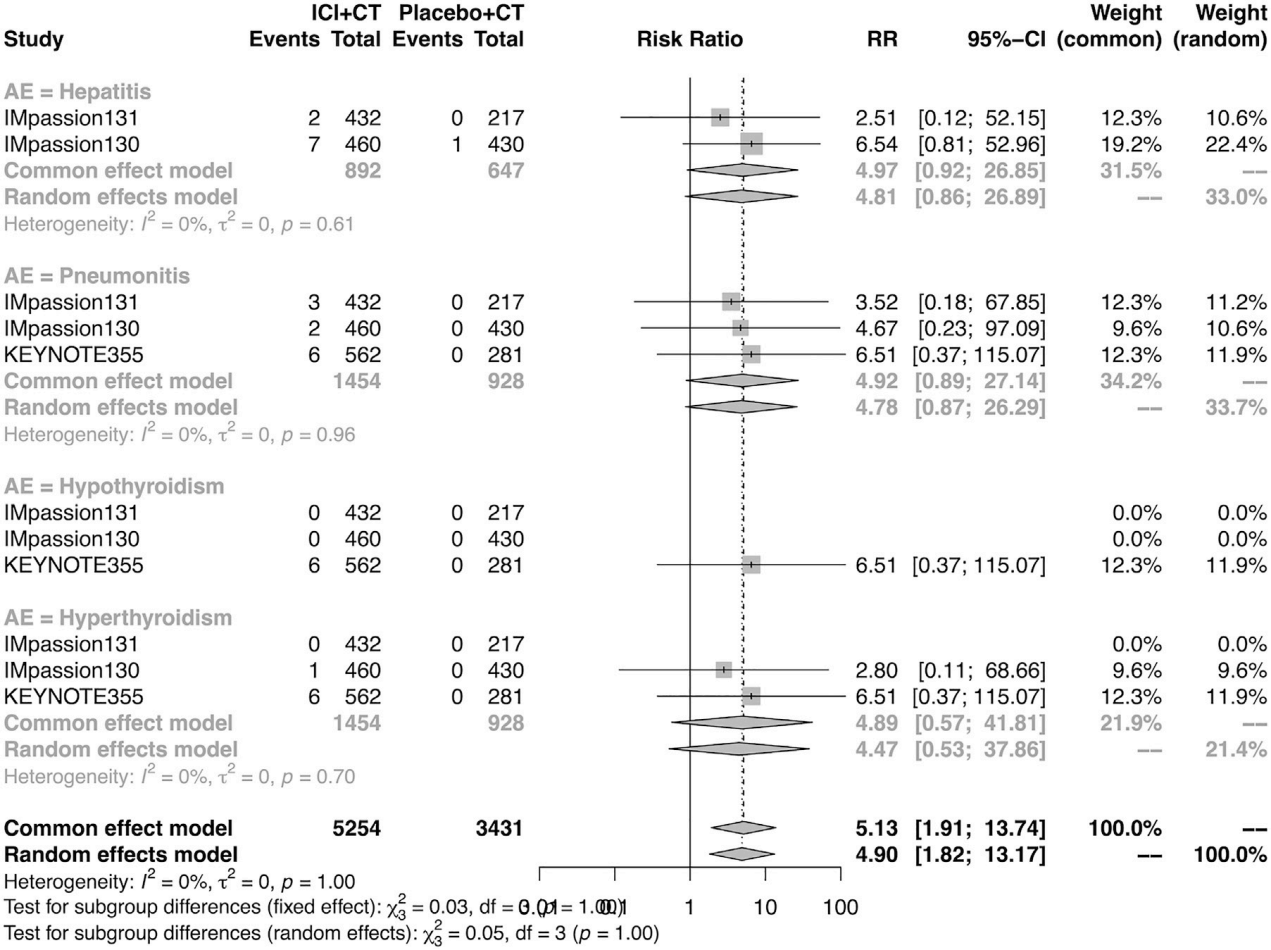
Pooled grade ≥3 AEs in adjuvant therapy clinical trials: severe cutaneous reactions. AEs, adverse events; RR, relative risk; 95% CI, 95% confidence interval; ICIs, immune checkpoint inhibitors; CT, chemotherapy.

**FIGURE 11:**
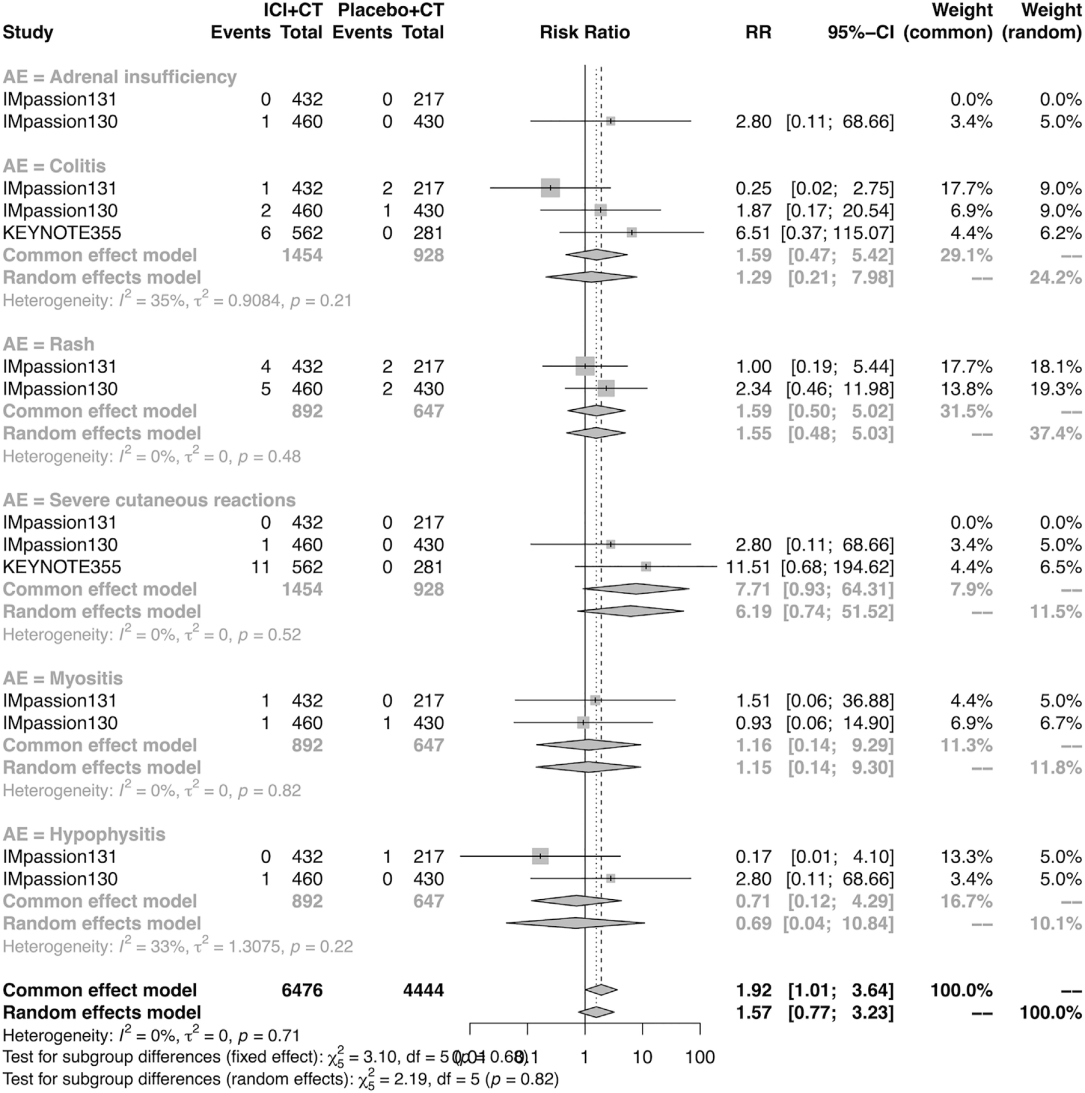
Pooled grade ≥3 AEs in adjuvant therapy clinical trials: severe cutaneous reactions. AEs, adverse events; RR, relative risk; 95% CI, 95% confidence interval; ICIs, immune checkpoint inhibitors; CT, chemotherapy.

The details of overall AEs reported in at least two studies in the adjuvant setting were analyzed in IMpassion 130, IMpassion 131, and KEYNOTE-355. The estimation for any grade AEs showed that the combination of ICIs and CT arm was associated with a higher incidence of hyperthyroidism (RR = 5.86; 95% CI 2.84–12.11, I^2^ = 0%), hypothyroidism (RR = 3.72; 95% CI 2.69–5.16, I^2^ = 45%), pneumonitis (RR = 8.35; 95% CI 2.90–24.04, I^2^ = 0%), and rash (RR = 1.26; 95% CI 1.08–1.47, I^2^ = 46%) with no heterogeneity (Figures 8, 9). No significant correspondence was found in adrenal insufficiency, colitis, hepatitis, hypophysitis, myositis, and severe cutaneous reactions in the adjuvant setting (Figures 8, 9). The estimation for grades more than three AEs showed that no significant correspondence was found in the increasing risk of adrenal insufficiency, colitis, hepatitis, hyperthyroidism, hypophysitis, hypothyroidism, myositis, pneumonitis, rash, and severe cutaneous reactions for combination strategy (Figures 10, 11).

### The detail of adverse events in the neoadjuvant setting

**FIGURE 12:**
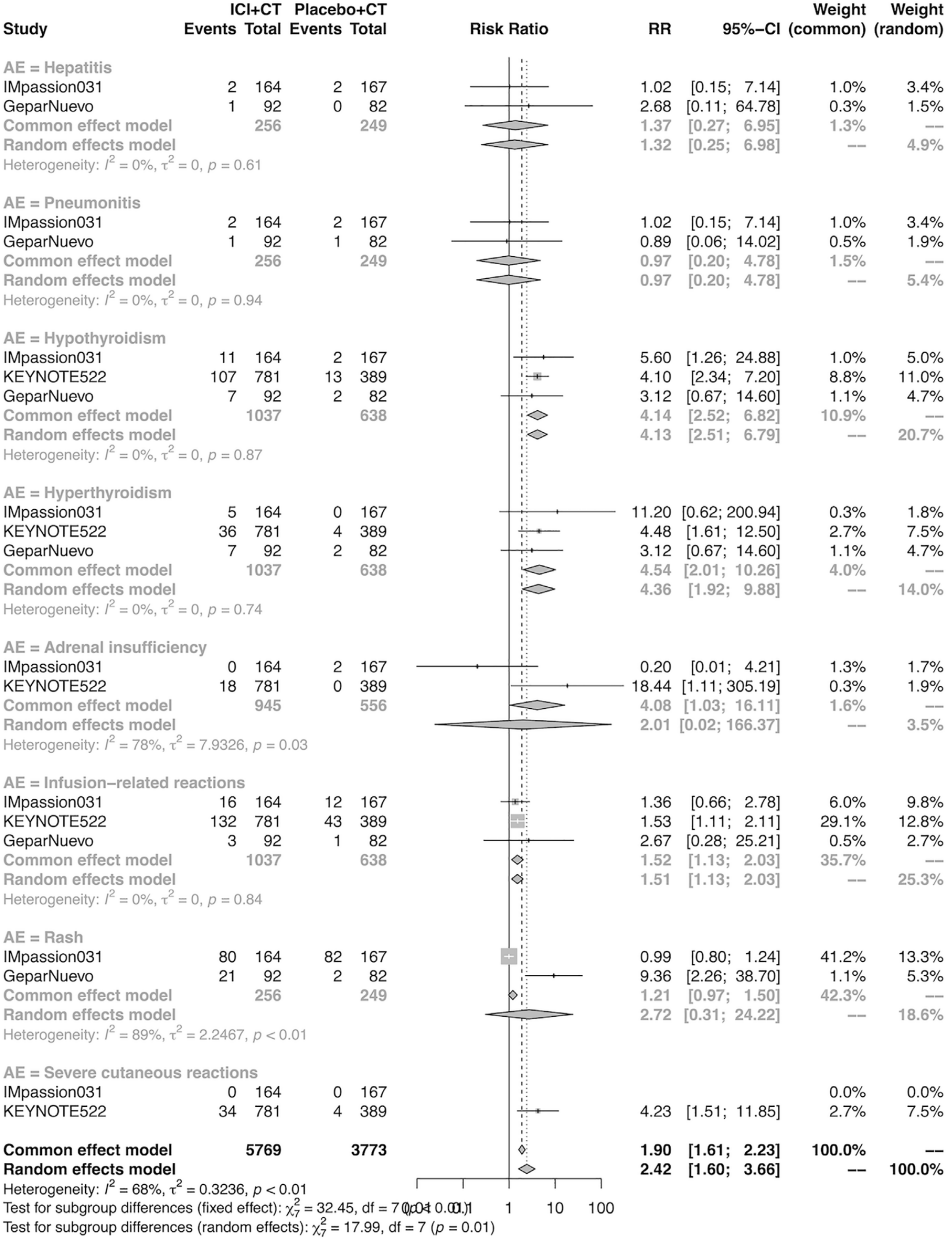
Pooled specific AEs in any grade in neoadjuvant therapy clinical trials. AEs, adverse events; RR, relative risk; 95% CI, 95% confidence interval; ICIs, immune checkpoint inhibitors; CT, chemotherapy.

**FIGURE 13:**
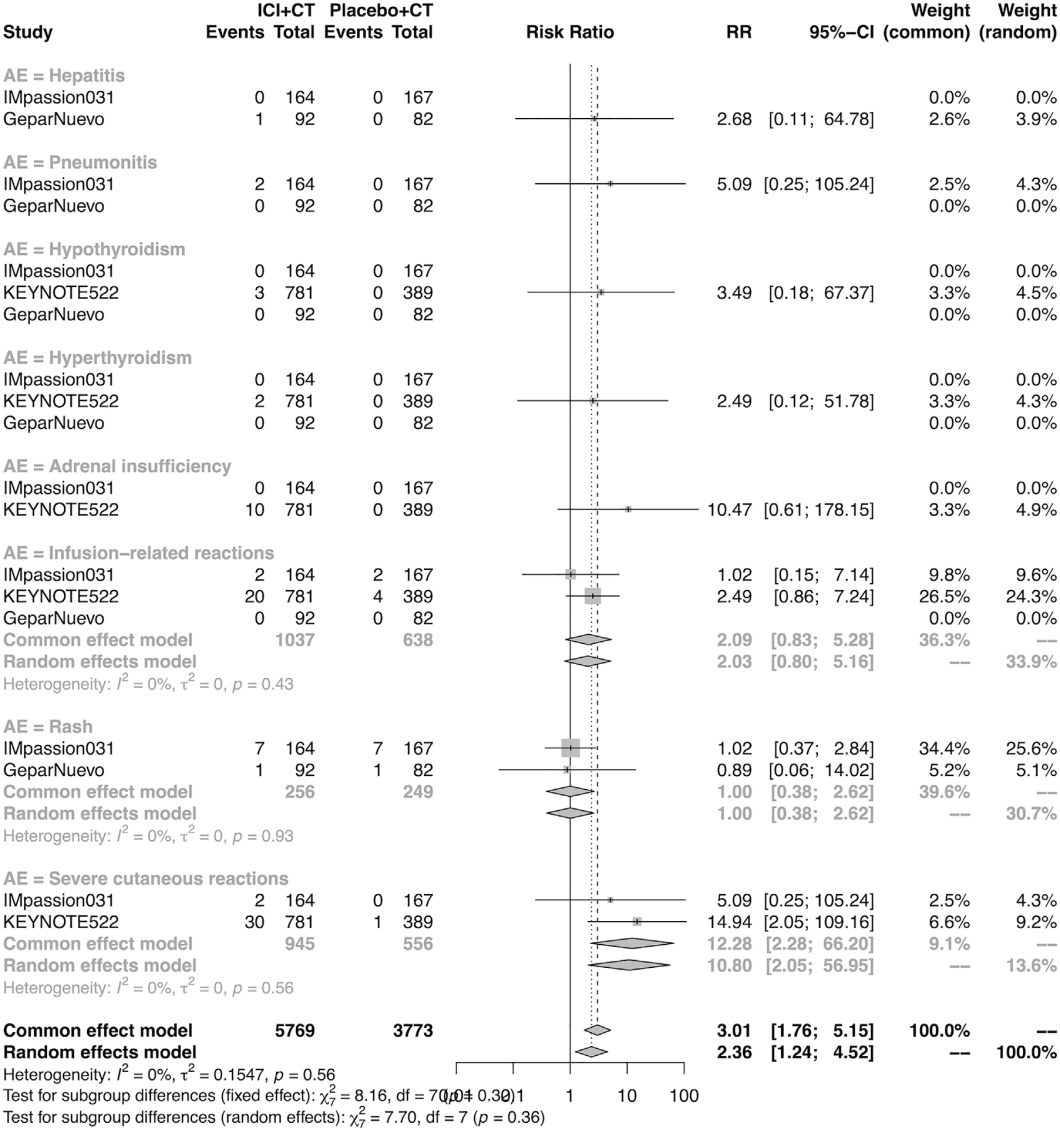
Pooled grade ≥3 AEs in neoadjuvant therapy clinical trials: severe cutaneous reactions. AEs, adverse events; RR, relative risk; 95% CI, 95% confidence interval; ICIs, immune checkpoint inhibitors; CT, chemotherapy.

The details of overall AEs reported in at least two studies in the neoadjuvant setting were analyzed in IMpassion 031, KEYNOTE-522, and GeparNuevo. The estimation for any grade AEs showed that the combination of ICIs and CT arm was associated with a higher incidence of hyperthyroidism (RR = 4.54; 95% CI 2.01–10.26, I^2^ = 0%), hypothyroidism (RR = 4.14; 95% CI 2.52–6.82, I^2^ = 0%), infusion-related reactions (RR = 1.52; 95% CI 1.13–2.03, I^2^ = 0%), and severe cutaneous reactions (RR = 4.23; 95% CI 1.51–11.85) with no heterogeneity (Figure 12). No significant correspondence was found in adrenal insufficiency, hepatitis, pneumonitis, and rash in the neoadjuvant setting (Figure 12). The estimation for grades more than three AEs showed that no significant correspondence was found in the increasing risk of adrenal insufficiency, hepatitis, hyperthyroidism, hypothyroidism, infusion-related reactions, pneumonitis, and rash, except for severe cutaneous reactions (RR = 0.08; 95% CI 0.02–0.44, I^2^ = 0%) for combination strategy (Figure 13).

### Quality assessment and publication bias

The risks of bias in included studies are summarized in Supplementary Figure S5. Based on the fact that the six studies were randomized, double-blinded, and with a specified analysis strategy and investigator assessment, all studies were considered at a low risk of selection, performance, detection, attrition, and reporting bias. The Egger’s test and funnel plot showed no evidence of publication bias (Supplementary Figure S6).

## Discussion

To our knowledge, this is the most comprehensive, largest, and up-to-date meta-analysis evaluating the efficacy and safety of ICIs in combination with chemotherapy as the first-line of adjuvant treatment in patients with metastatic TNBC and neoadjuvant treatment in patients with early-stage TNBC. There are six published RCTs with discordant findings in this field (IMpassion131, IMpassion130, KEYNOTE-355, IMpassion 031, KEYNOTE-522, and GeparNuevo). Our study aimed to push the dense fog aside and clarify the efficacy and safety of the combination of PD-1/PD-L1 ICIs and chemotherapy applied to adjuvant and neoadjuvant treatments.

Our findings confirmed that neoadjuvant ICIs plus CT combination improves the pCR rate of early-stage TNBC patients in the ITT group by 29%, and we also verified that adjuvant combination therapy benefits the PFS in unresectable locally advanced or metastatic TNBC in both ITT and PD-L1-positive groups. Driven by the PD-L1 status, this PFS benefit enables a 29% relative risk of progressive reduction in the PD-L1-positive population. However, no benefit was found in terms of OS and TTD in both ITT and PD-L1-positive populations under the adjuvant setting, and no benefit for any-reason death in the PD-L1-positive population was found.

In terms of the safety of this combination strategy, though the general profile was consistent with the published side effects of the regimen in individual studies, discrepancies were shown between adjuvant and neoadjuvant settings. There is no doubt that the idiosyncratic AEs can occur in the experimental group due to the use of immunosuppressants. However, our analysis shows that the side effects of this combination therapy are overall safe and tolerable.

In general, the combination strategy did not increase the risk of any AEs in the neoadjuvant setting. Although the experimental group had more adverse reactions at any grade in the adjuvant setting, there was no difference concerning the AEs above grade III. In detail, in addition to increasing the risk of hypothyroidism, hyperthyroidism, pneumonia, and rash at any grade in the adjuvant setting, there is an increasing risk of hypothyroidism, hyperthyroidism, infusion-related reactions, and severe cutaneous reactions at any grade in the neoadjuvant setting, as well as the risk of severe cutaneous reactions above grade III in both settings, there was no difference existing in other adverse events. Hypothyroidism is widely acknowledged as the most common immune-related endocrine toxicity, which occurs in approximately 30%–40% of anti-PD-1/PD-L1-treated patients (Larkin et al., 2019; Wright et al., 2021). Additionally, anti-PD-1/PD-L1 is a more potent type of hypothyroidism-inducing ICI treatment than anti-CTLA4 and a less potent treatment than the combined type (de Filette et al., 2019). Hyperthyroidism is another symptom of immune-related endocrine dysfunction, and a previous study reported that the risk of hyperthyroidism was significantly higher among patients treated with PD-1 than that with PD-L1 ICIs and among those treated with pembrolizumab compared with nivolumab (Barroso-Sousa et al., 2018; Chiloiro et al., 2022), and this was not the same in terms of hypothyroidism (Barroso-Sousa et al., 2018).

Specifically, it should be noted that in the adjuvant setting, the combined strategy produced a 33% statistically significant relative increase in the risk of severe AEs but not grades more than three AEs and death events. This may result from the fact that although the overall number of high-grade AEs in the two groups is roughly the same, the AEs caused by the combination are concentrated in more serious events but not death. In terms of that in the neoadjuvant setting, the analysis cannot be performed due to a lack of relative data. Therefore, based on current evidence, the safety of combination therapy is generally mild and well-tolerated, but we can never be careless about serious events, especially in the adjuvant setting, which needs more in-depth research and discussion to reduce and control them.

The side effect profiles of the neoadjuvant therapy group and adjuvant therapy group were almost the same, but there were still some differences. For example, there were more dermatitis and infusion-related reactions in the neoadjuvant treatment group, while there was a high rate of pneumonia in the adjuvant treatment group. One thing that should be clarified is that the analysis was not performed due to insufficient infusion-related reaction data in the adjuvant treatment group. According to the existing evidence, it can be speculated that the difference in pneumonia may be related to the baseline characteristics of the population to some extent: most patients in the neoadjuvant treatment group are in the early stage, with low ECCG scores and no lung metastasis, while patients in the adjuvant treatment group are in the advanced stage, with high ECOG scores, and nearly half of the patients have lung metastasis. The intervention of ICIs may be more likely to affect this part of the population. Additionally, compared with patients in other clinical trials, patients in the GeparNuevo have a higher risk of rash, which may be because GeparNuevo is the only study using durvalumab as an ICI, and rash/dermatitis was reported to be one of the most common adverse events among patients receiving durvalumab (Ribas et al., 2020; Ghebeh et al., 2021; Fahmy et al., 2022; Naidoo et al., 2022). Additionally, in an unresectable stage III NSCLC durvalumab monotherapy setting, Naidoo J et, al. reported that any-grade dermatitis/rash was related to the shortest median onset time among the non-pneumonitis immune-mediated AEs since the beginning of durvalumab [37.0 days [range, 6–111 days); *n* = 9], but with the longest resolution time [104.0 days (range, 17–326 days); *n* = 11]. Therefore, in a neoadjuvant setting, rash symptoms should be paid more attention to at the initiation of the durvalumab and should be given more time to recover.

Yunhai Li et, al. and Ji Qiao et, al. conducted meta-analyses regarding the efficacy and safety of the combination of ICIs and chemotherapy among TNBC patients (Ji et al., 2021; Li et al., 2021), which reported similar results as ours. Additionally, the differences lie mainly in the following aspects: 1) our study included and analyzed both neoadjuvant and adjuvant studies, while Yunhai Li et, al. merely focused on the adjuvant setting and Ji Qiao et, al. only paid attention to the adjuvant setting; 2) we analyzed and discussed the discrepancies of adverse events in detail between these two settings to sort out the adverse events that require additional attention due to the different combined strategies of ICIs; 3) subgroup analyses of OS in adjuvant setting according to age, race, baseline disease status, metastatic sites, neoadjuvant therapy, previous treatment, and so on were well-performed by Ji Qiao et, al, through which they reported that the Asian patients, patients with locally advanced disease, and patients with brain metastases might not benefit from the addition of ICIs (Ji et al., 2021).

This meta-analysis’s limitations are as follows. First, we did not perform analysis stratified by age, CT regimen, ICI regimens, ECOG, number of metastatic sites, and so on. Second, PD-L1 was assessed in different cells in these studies: PD-L1 was assessed in immune cells in IMpassion 131, IMpassion 130, and IMpassion 031, whereas PD-L1 was assessed in iTILs (tumor cells, lymphocytes, and macrophages) in KEYNOTE-355, KEYNOTE-522, and GeparNuevo. In addition, the PD-L1 assessment techniques were different: IMpassion 131 and IMpassion 130 were assessed by VENTANA PD-L1 (SP142) immunohistochemical testing, while KEYNOTE 355, IMpassion 031, and KEYNOTE 522 were assessed using the IHC 22C3 pharmDx assay and characterized by the CPS, and the GeparNuevo used the Ventana SP263 antibody to assess. Therefore, we should proceed with extra caution when interpreting the findings of PD-L1-positive populations in these studies. Third, since the adverse events were not homogenous among these six studies, the safety analysis was performed only for those adverse events reported in at least two studies. Fourth, our analysis is based on only three studies each in adjuvant and neoadjuvant settings, which could be inadequate for visual or statistical examination of publication bias. Fifth, due to the lack of OS data in the KEYNOTE 355 study, the pooled OS analysis in the adjuvant setting only included IMpassion 131 and IMpassion 130. Sixth, due to the extremely low incidence rate of some of the detailed AEs in the neoadjuvant setting, the pooled evaluation should be carefully interpreted. In addition, this study may be subjected to any errors and biases from the original investigators, thus the meta-analysis findings are generalizable only to patients eligible for these clinical trials.

## Conclusion

Our meta-analysis demonstrated that a combination of ICIs and CT can effectively improve the pCR rate in early-stage TNBC patients in the neoadjuvant setting, and it also benefits a better PFS in untreated, unresectable, locally advanced, or metastatic TNBC patients in the adjuvant setting. The PD-L1-positive status is regarded as a driven factor for better efficacy. The safety profiles of the experimental arm are generally good and tolerable compared with the control arm, but it is still essential for clinical physicians to pay close attention to those severe adverse events. To summarize, based on the current evidence concerning efficacy and safety data, the combination of ICIs and CT regimen can be recommended in early-stage TNBC patients in the adjuvant setting and untreated unresectable locally advanced or metastatic TNBC patients in the adjuvant setting, especially in PD-L1-positive population.

## Data Availability

The raw data supporting the conclusion of this article will be made available by the authors, without undue reservation.
