# Supplementary figures and images for: Efficacy and safety of PD-1 and PD-L1 inhibitors combined with chemotherapy in randomized clinical trials among triple-negative breast cancer
Source: Front Pharmacol. 2022 Sep 16;13:960323. doi: 10.3389/fphar.2022.960323 (PMC9523473; doi:10.3389/fphar.2022.960323)

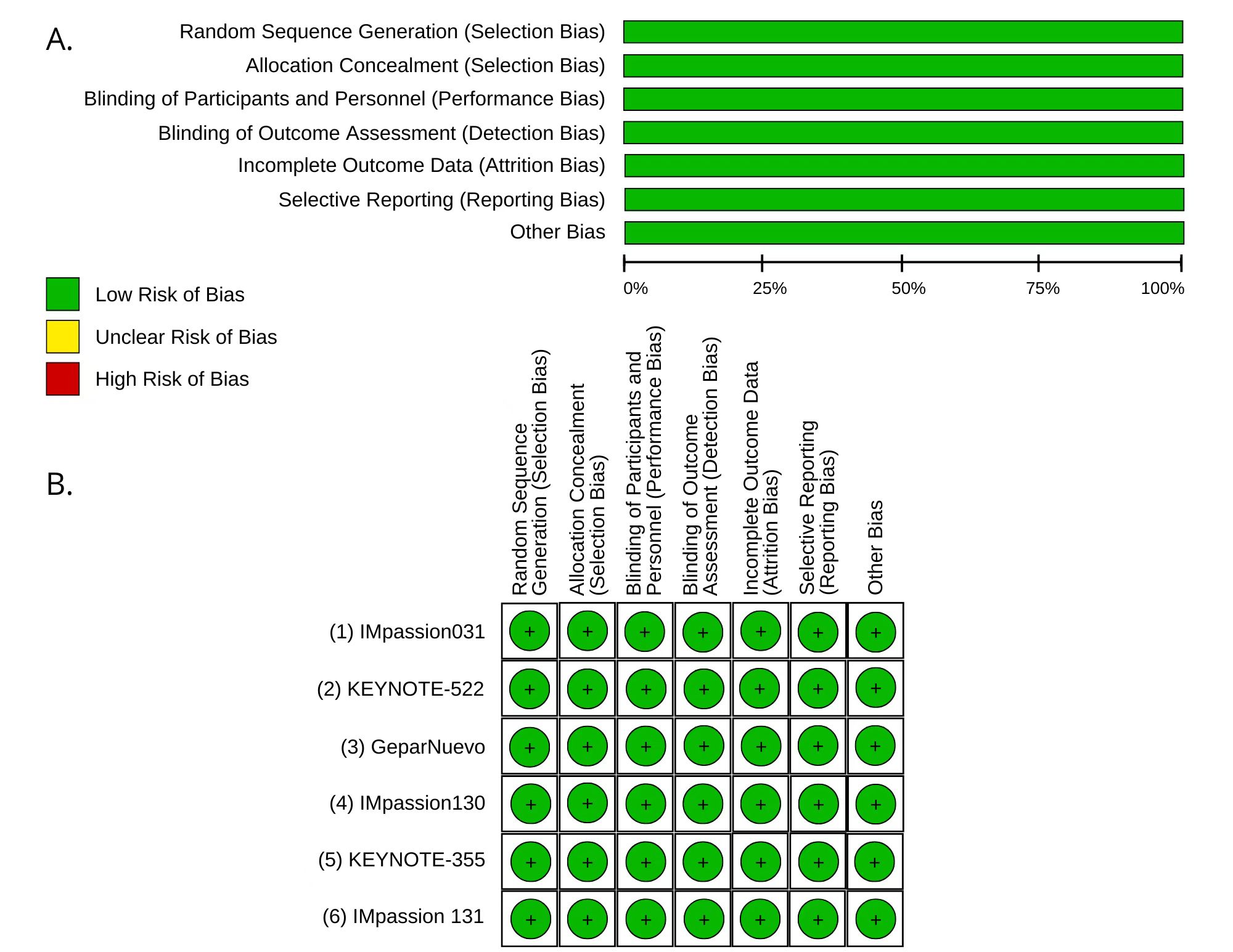

Supplement: Supplementary file 1 [file Image1.JPEG]

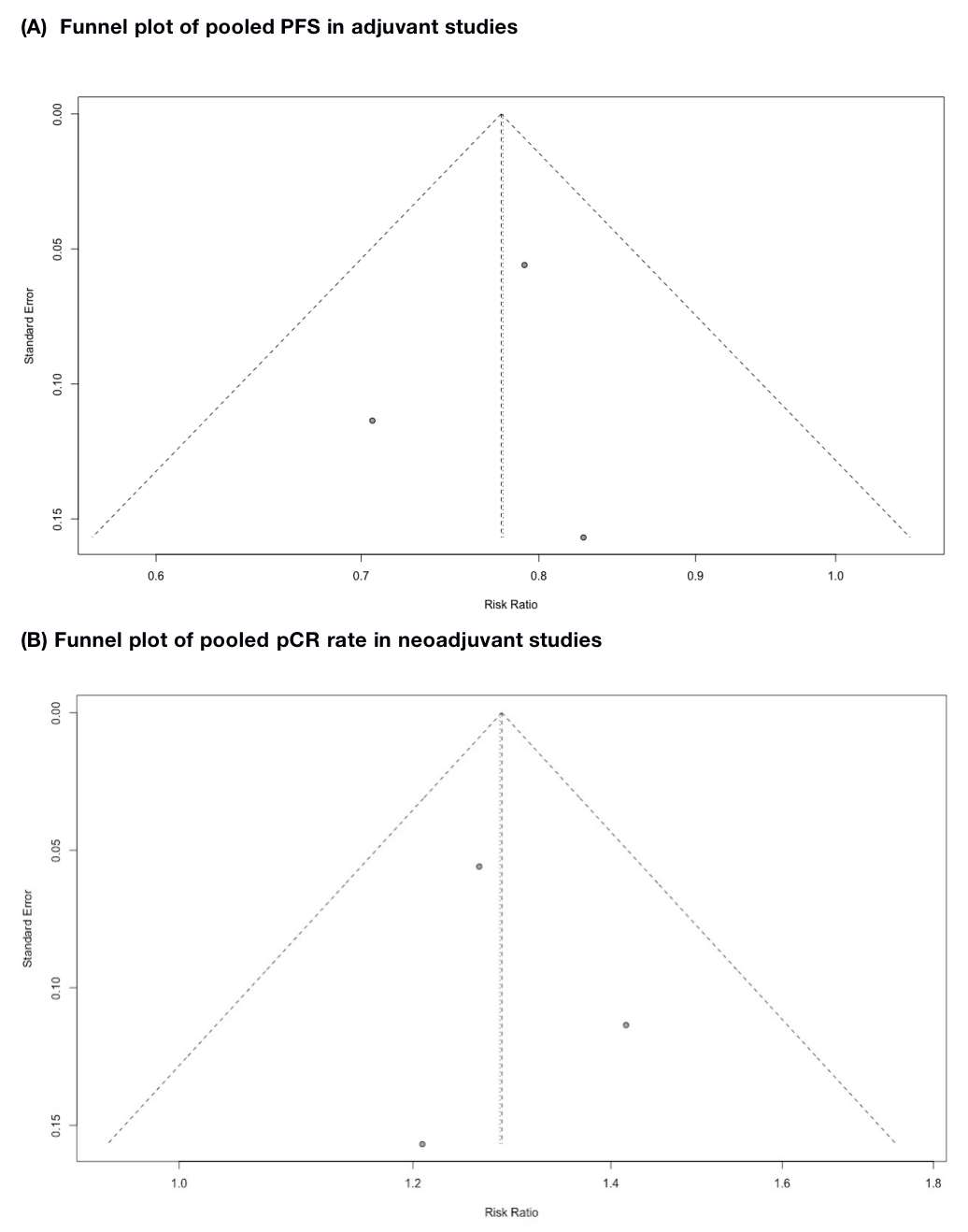

Supplement: Supplementary file 2 [file Image2.PNG]
